# Current News

**Published:** 1889-02

**Authors:** 


### Current News.

—Canada is to have a dental journal with Dr. W. Geo. Beers as editor.

Dr. S. A. White thinks a dentist should make every effort possible to preserve the deciduous teeth until nature has thrown them off.

Dr. Carpenter believes that fully 90 per cent. of the diseases of the eye and ear, catarrh, rheumatism and palsy, might be traced to diseases of the teeth and mouth.

Dr. Gordon, a physician, of Dalton, Ga., states that an ulcerated tooth may produce conditions resembling cancer, and that neuralgic eye; or earache may be caused by carious teeth.

Dr. R. W. Thornton uses "'Probes and Patience' in the treatment of devitalized teeth, and pumps carbolic acid through a fistulous opening." [Certainly the doctor is standing on a sound foundation when he commences with "patience," and it would be well if all would emulate his method.—Ed.]

Dr. Holland takes the impression of a very difficult case in two pieces, taking the innner portion first. Making grooves in this piece he oils and varnishes it, and then replaces in the mouth and takes the outer portion. Taking them out separately, they are placed together again, out of the mouth, and the cast made as usual.

The Georgia State Dental Soc. in discussing the relative frequency of fatal results under the capital operations of general surgery, and the minor operations of dental surgery, came to the conclusion, that pain being the antidote for effects of anæsthesia, the greater danger in trifling operations is that there is not sufficient pain to counteract the effects of the anæsthetic, and therefore it should not be used in the smaller operations of dental surgery.

We have been requested to give the formula for Golden's Liquid Beef Tonic that we recommended in our November number, which is as follows:

| | |
|---|---|
| 20 per cent. saccharine matter | 20 |
| 25 per cent. glutinous or nutritous matter obtained in the condensation of the beef | 25 |
| 25 per cent. spirits rendered non-injurious to the most delicate stomach by the extraction of the fusel oil | 25 |
| 30 per cent. of an aqueous solution of several herbs and roots, among which are most discernible Peruvian and Calisaya barks | 30 |

With a sufficient quantity of the soluable of citrate of iron to represent 2 grs. to wine-glassful of the preparation.

Dr. J. Y. Crawford says: "The dental pulp is not a peripheral extremity of nerve; but a ganglion—a bundle of nerves. It excites nervous reflex pain, and is in action like the iris, to which it is wonderfully near of kin; but is not so amenable to treatment as that delicate organ.

Dr. W. H. Morgan, Nashville, Tenn., recommends ammonia as the best thing to dissolve away deposits on artificial dentures, claiming that soap does not meet the requirements.

### BENZOIC SULPHIDE OF SODIUM AS AN ANTISEPTIC.

E. Heckel, says the *Comptes Rendus Bullet. Commerc.*, prepares benzoic sulphide of sodium, or natriumsulfibenzoat, as it is called in Germany, by dissolving a large quantity of benzoic acid in a concentrated solution of sulphide of sodium. This preparation is perfectly harmless, even when given in large doses. It is easily soluble in water, and contains the antiseptic properties of its two principal constituents.

According to the experiments of Heckel and other surgeons, among whom we may mention Professor Fontan, of Toulon, it is a most valuable dressing for wounds, when used as a wash or upon a bandage. Four to five grammes (30 to 45 grains) of the preparation are used to every quart of water. Its antiseptic properties are very active and promote healing. Heckel considers it to be equal to carbolic acid, and superior both to sublimate and iodoform, as it is not poisonous, whereas the former is, and because it is free from any disagreeable effects like those of the latter.—*Therapeutic Gazette*, March, 1888.

---

On the influence of the wisdom teeth in process of eruption and disease in bringing about affections of the throat and lungs, Dr. J. Y. Crawford has made many interesting observations. He states that tonsilitis, inflammation of the mucous membrane lining the respiratory track, and even phthisis pulmonalis are frequently traceable to the difficult eruption of the wisdom teeth. In many cases of throat affections the primary lesion is of a traumatic nature, the closing of a cusp on the outer jaw pinching and hardening the tissues, and from that indurated structure a line of inflammation passes back to the tonsils, which in turn fail to secrete their lubricating fluids; hence throat troubles, chafing, ulceration, etc., becoming a permanent disability.

Dr. J. Y. Crawford states that the removal of an offending wisdom tooth may prove a factor in averting premature decline.

Dr. W. H. Morgan, Nashville, Tenn., emphasizes the importance of drilling out root canals before filling, especially in middle-aged patients, when the entrance of the canal is apt to be partially closed by secondary dentine. He objects to the oxychloride filling as becoming porous, and either letting offensive gases pass through or absorbing them; and also states that the end of the root should always be reached through the fistula. It will often be found roughened by dissolved dentine, and this should be smoothed off.

Dr. J. Y. Crawford believes that the neglect of the preservation of the natural teeth, and the unnecessary extraction of teeth, often for twenty-five cents apiece, are prime factors in causing the shortening of life in the United States.

Meeting the objection that bridge-work was injurious to the adjoining teeth, Dr. J. Y. Crawford states that he thinks movable plates more open to that objection than modern bridge-work properly applied.

Dr. H. S. Colding is not in favor of replantation, claiming that if a tooth was too frail to fill, he would rather cut it off and crown it. He treats the root-canals of dead teeth with a paste of iodoform and wood creosote, stopping with Gilbert's patent filling material.

In filling roots with oxychloride of zinc, Dr. S. Holland, Atlanta, Ga., inserts a flattened gold wire, bevelled at the end going to the apex, which closes its opening and allows none of the material to pass through.

In the recent Georgia State Dental Convention, Dr. Main stated that his (medical) profession, as a rule, paid too little attention to the condition of the mouth and teeth as a factor in disease; the effects of imperfect mastication, decomposing food, etc., around the teeth on the general health; and readily admitted (though it was not generally recognized) that many forms of throat and lung trouble are traceable to lesions in and around the teeth, many cases of so-called "mumps," sarcoma, cancer, etc., having their origin in diseases of the dental organs.

In the treatment of devitalized deciduous teeth, Dr. Crawford considers it the best practice not to extract, but to keep the teeth non-antagonizing and isolated, and they will exfoliate. Permanent dentition will not be interfered with, although it may be retarded.

Dr. H. H. Johnson, Hawkinsville, Ga., uses oxychloride of zinc for filling root-canals, claiming it to be a disinfectant which has a drying effect on dentine. He uses engenol exclusively for checking putrefactive changes, it having no escharotic qualities; non-irritant if pumped into the apex, and not coagulating albumen; also having no objectionable odor, while possessing all the good qualities of carbolic acid and none of its unpleasant ones.

Dr. Abbott does not think implanted teeth are retained in their position either by anchylosis or gumphosis, any more than a nail in a board is retained by any peculiar form of attachment. The inflammation induced in the surrounding bone in the process of drilling out, however, may produce granulations, which force their way into the irregularities on the surface of the implanted tooth.

In reference to the form of attachment, he thought the sharp resonant sound given out by an implanted tooth was proof of other than mere fibrous attachment.

The operation of sponge grafting, Dr. Taft thinks, throws light on question of attactment of the tooth in implantation. The cementum of the tooth is structurally ready, like the sponge, to receive the plasma thrown out, which displaces the organized material, and replaces it by living tissues. He says no other hypothesis can explain it; but it is a theory which needs demonstration and proof.

---

### INCREASING THE ANTISEPTIC POWER OF CRUDE CARBOLIC ACID.

Although it has been demonstrated by Koch that pure carbolic acid is one of our best antiseptics, we have been unable to utilize the crude acid on account of uncertain and slight disinfectant power. This was due to the fact that crude carbolic acid is nearly insoluble in water and watery fluids. Laplace has recently found that the addition of acids, particularly sulphuric, to crude carbolic acid, of different strengths, considerably increases its solubility. Still better results were obtained by mixing 25 per cent. crude carbolic acid with an equal quantity of concentrated crude sulphuric acid. A thick, syrupy, homogeneous, dark-brown mass resulted, which dissolved readily and completely in water. It was further found that combinations of these two acids possess great disinfectant powers. Anthrax spores, immersed in 2 per cent. solutions of the combined acids, were destroyed in seventy-two hours; and in forty-eight hours when a 4 per cent. solution was employed. In this respect sulpho-carbolic acid surpasses a 2 per cent. solution of pure carbolic acid and crealin, the newly introduced antiseptic, and is only inferior to a 5 per cent. carbolic solution and a 1 to 1000 acid solution of corrosive sublimate. An equally cheap, attainable and effective disinfectant is not known.—Translated from *Deut. Medicin. Wochenschr.*, No. 7, 1888.—*Jour. of Surgery and Antiseptics.*

